# Effects of target-amplitude and background-contrast uncertainty predicted by a normalized template-matching observer

**DOI:** 10.1167/jov.23.12.8

**Published:** 2023-10-25

**Authors:** Can Oluk, Wilson S. Geisler

**Affiliations:** 1 Center for Perceptual Systems and Department of Psychology, University of Texas at Austin, Austin, TX, USA; 2 Laboratoire des systèmes perceptifs, Département d’études cognitives, École normale supérieure, Université PSL, Paris, France; 3 Center for Perceptual Systems and Department of Psychology, University of Texas at Austin, Austin, TX, USA

**Keywords:** visual detection, spatial vision, uncertainty, ideal observer analysis, contrast gain control

## Abstract

When detecting targets under natural conditions, the visual system almost always faces multiple, simultaneous, dimensions of extrinsic uncertainty. This study focused on the simultaneous uncertainty about target amplitude and background contrast. These dimensions have a large effect on detection and vary greatly in natural scenes. We measured the human performance for detecting a sine-wave target in white noise and natural-scene backgrounds for two levels of prior probability of the target being present. We derived and tested the ideal observer for white-noise backgrounds, a special case of a template-matching observer that dynamically moves its criterion with the background contrast (the DTM observer) and two simpler models with a fixed criterion: the template-matching (TM) observer and the normalized template-matching (NTM) observer that normalizes template response by background contrast. Simulations show that, when the target prior is low, the performance of the NTM observer is near optimal and the TM observer is near chance, suggesting that manipulating the target prior is valuable for distinguishing among models. Surprisingly, we found that the NTM and DTM observers better explain human performance than the TM observer for both target priors in both background types. We argue that the visual system most likely exploits contrast normalization, rather than dynamic criterion adjustment, to deal with simultaneous background contrast and target amplitude uncertainty. Finally, our findings show that the data collected under high levels of uncertainty have a rich structure capable of discriminating between models, providing an alternative approach for studying high dimensions of uncertainty.

## Introduction

Most of the essential natural tasks for survival (finding food, noticing predators, finding a potential mate) require the detection of objects of interest (targets) in the visual scene. Some of these natural tasks involve detection of specific individual targets (a friend, a pet, the car keys), and some involve detection of a target belonging to a large category (a human, a dog, a car). Under natural conditions, detection is difficult and complicated because there are high levels of stimulus variability. For example, targets are rarely in the same position, rarely have the same three-dimensional orientation, and rarely have the same illumination. In addition, the specific context (background) in which the target is located is also rarely the same. In other words, visual systems must evolve and/or learn to perform detection under high levels of external stimulus variability, which we also refer to here as “extrinsic uncertainty.”

External stimulus variability is present in every possible detection task. At the minimum, the stimulus varies in each trial due to photon noise and the random probability of a fixed target being present or absent. In this minimum-variability task, the specific stimuli in the two categories (present or absent) are held fixed (except for photon noise) within a block of trials, allowing a rational observer (in the sense of signal detection theory) to adopt a fixed decision criterion for that block. In these tasks, performance is usually limited primarily by internal factors, including various sources of internal noise and nonlinearity. A great deal about human detection mechanisms has been learned from these minimum-variability tasks. However, as mentioned above, under natural conditions there is a great deal more external variability from one occasion (trial) to the next. Thus, to better understand the effects of stimulus uncertainty many studies have included additional sources of external variability.

A classic example is detection of targets in noise backgrounds (Burgess, Wagner, Jennings, & Barlow, 1981). On each trial in a block, a different random sample of noise background is presented, and on half of the trials a fixed-amplitude target is added to the noise background. In each block, the noise has some nominal (average) contrast. Under these stimulus conditions, the detection threshold increases in proportion to the nominal noise contrast and is limited by both the external noise and internal factors. Similar results have been obtained with random samples of natural background (Sebastian, Abrams, & Geisler, 2017). However, in these studies, unlike under natural conditions, the background contrast and the target amplitude were blocked. It has previously been found that the contrast at one location is largely uncorrelated with the contrast at locations only a couple of degrees away (Frazor & Geisler, 2006). Thus, the local background contrast during each fixation will likely differ from the contrast at the same retinal location during the previous fixation. Therefore, the visual system almost always faces trial-by-trial variability in background contrast. Similarly, there are multiple sources of variation in the target amplitude. Sometimes within the same target category, different individual members share the same visual pattern but vary in amplitude. Second, the same individual member's amplitude might also vary due to variations in the illumination. Therefore, even when searching for a fixed visual pattern, the visual system often faces uncertainty about the target amplitude.

In sum, simultaneous target-amplitude and background-contrast variability is almost always present under naturalistic conditions. This fact motivated us to measure detection performance in noise backgrounds when the target amplitude and noise contrast were randomly varied on each trial. Ignoring photon noise, there were three dimensions of variability in each trial: the spatial pattern of the background, the contrast of that pattern, and the target amplitude. To help distinguish among models, we also varied the prior probability of the target being present.

Most previous studies of human detection have focused on tasks with just one or two dimensions of external variability. This is a sensible strategy because it reduces the complexity of the experimental design, data analysis, and theory. For example, there is extensive literature on visual search (for reviews, see Eckstein, 2011; Wolfe, 2015), which, by definition, involves target-location uncertainty (Eckstein, Thomas, Palmer, & Shimozaki, 2000). Specifically, a number of studies have been aimed at understanding the effect of target-location uncertainty in isolation (Cohn & Lasley, 1974; Swenson & Judy, 1981; Davis, Kramer, & Graham, 1983; Cohn & Wardlaw, 1985; Solomon, Lavie, & Morgan, 1997; Foley & Schwartz, 1998; Eckstein et al., 2000; Nachmias, 2002). Most of the other dimensions of uncertainty have also been studied in isolation. There have been studies focusing on uncertainty about target orientation (Doehrman, 1974; Ukkonen, Rovamo, & Näsänen, 1995; Silvestre, Cavanagh, Arleo, & Allard, 2017), target phase (Burgess & Ghandeharian, 1984; Howard & Richardson, 1988), target size (Judy, Kijewski, Fu, & Swensson, 1995; Judy, Kijewski, & Swensson, 1997; Meese, Hess, & Williams, 2005; Foley, Varadharajan, Koh, & Farias, 2007; Meese & Summers, 2012) and target spatial frequency (Davis & Graham, 1981; Davis et al., 1983; Yager, Kramer, Shaw, & Graham, 1984; Hübner, 1996a; Hübner, 1996b; Ohtani & Mori, 2002). Some of these studies also include noise backgrounds (background pattern variability). There have also been some studies focusing on background pattern variability (Ahumada & Beard, 1997; Beard & Ahumada, 1999). However, there have been relatively few studies involving simultaneous dimensions of uncertainty (Eckstein, Whiting, & Thomas, 1996; Eckstein, Ahumada, & Watson, 1997; Wilson & Manjeshwar, 1999; Eckstein & Abbey, 2001; Park, Clarkson, Kupinski, & Barrett, 2005).

The most common method for studying the effects of extrinsic uncertainty is to select a range of levels along the uncertainty dimension, measure performance separately for each of those levels, and then compare those measures of performance to the performance measured when the levels on each trial are randomly selected from the entire set (for reviews, see Cohn & Lashley 1986; Carrasco, 2011; Eckstein, 2011; Wolfe, 2015). Although this is a useful and practical approach for individual dimensions of variability, it is less practical for multiple dimensions of variability because of the need to measure performance for all combinations of levels along the multiple dimensions. Furthermore, the number of trials required to obtain reliable data for the individual levels is higher because a substantial number of practice trials (or larger blocks) are necessary for participants to adjust their decision strategy for the specific combination of levels in each block. Also, more trials are required because the proportion of correct rejections must also be estimated for each combination of levels. A more practical approach is to make measurements only in the full uncertainty condition, where the levels are randomly selected on each trial. These data have been used to discriminate among various computational hypotheses for the detection under uncertainty (Eckstein & Whiting, 1996; Bochud, Abbey, & Eckstein, 2004; Castella et al., 2008; Castella et al., 2009; Meese & Summers, 2012; Han & Baek, 2020). Specifically, when there are multiple dimensions of uncertainty, a rich set of hits and correction rates is produced that can be used to discriminate among alternate models. Thus, we measured human detection performance in white noise and natural backgrounds for simultaneous target-amplitude and background-contrast variability to test among four model observers embodying different computational hypotheses.

We derived and tested the ideal and three sub-ideal observers. The ideal observer (IO) is a relatively simple extension of the classic template-matching observer that dynamically varies the decision criterion based on the estimated background contrast. The first suboptimal model observer is the classic template-matching (TM) observer. It does not dynamically vary its criterion but has a single fixed criterion applied regardless of background contrast; however, it is the standard observer for explaining performance when there is low uncertainty (Burgess et al., 1981; Sebastian et al., 2017). Thus, it represents the simplest (null) hypothesis: The underlying visual mechanisms under high uncertainty are the same as those under low uncertainty. The second plausible suboptimal candidate is the normalized template-matching (NTM) observer, which is constructed by normalizing the template response by the estimated level of background contrast. Normalizing responses by the estimated contrast (i.e., contrast gain control) is already a known property of neurons in the primary visual cortex (Albrecht & Geisler, 1991; Heeger, 1992; Carandini & Heeger, 1994; Carandini, Heeger, & Movshon, 1997; Cavanaugh, Bair, & Movshon, 2002). Therefore, it is a simple, biologically plausible extension of classic template matching. The third model observer was one where the criterion can be changed in an arbitrary way based on estimated background contrast. We refer to this as the dynamic template-matching (DTM) observer, which includes the ideal observer as a special case.

When we ran simulations of three of the model observers (IO, TM, NTM, DTM), we discovered that varying the prior probability of the target being present should be very useful for discriminating among the model observers. Under naturalistic conditions, the prior probability of a target being present can vary drastically from one environment to another. In general, the likelihood of a specific target being present at any random fixation location is extremely low; for example, the likelihood of a fork being present at any random fixation location in a home is almost zero. However, the likelihood might be higher for some rooms (the kitchen) and even higher in some confined regions (the kitchen table). The computations in the visual system must take into account, to at least some degree, the prior probabilities of targets being present. We found that the performance of the three model observers was much more discriminable when the target prior probability was low than when it was 0.5 (the typical value in detection and discrimination tasks).

In the first experiment, we measured human performance for detecting additive targets in white noise, when background contrast and target amplitude were randomly varying over a wide range, for different prior probabilities of target present (0.2 and 0.5). In the second experiment, we measured human detection performance with natural-scene backgrounds and tested the same model observers (although none of them was strictly ideal). Although the simple TM observer explains human performance in various basic visual detection experiments under low extrinsic uncertainty (Burgess et al., 1981; Sebastian et al., 2017; for reviews, see Cohn & Lasley, 1986; Geisler, 2003), we found that it fails to explain human performance in both noise and natural backgrounds under simultaneous target-amplitude and background-contrast uncertainty. We found that both the DTM and NTM observers explained the data well. Thus, the visual system might utilize dynamic criteria based on estimated contrast or normalize template responses by the estimated contrast, or a combination of the two.

More generally, our results demonstrate how the rich pattern of hits and correct rejections in only a single full uncertainty condition can provide sufficient data for distinguishing among model observers. Our results also demonstrate the potential value of manipulating the prior target present for distinguishing among model observers.

## Theory of detection under target and background uncertainty

**Figure 1.**
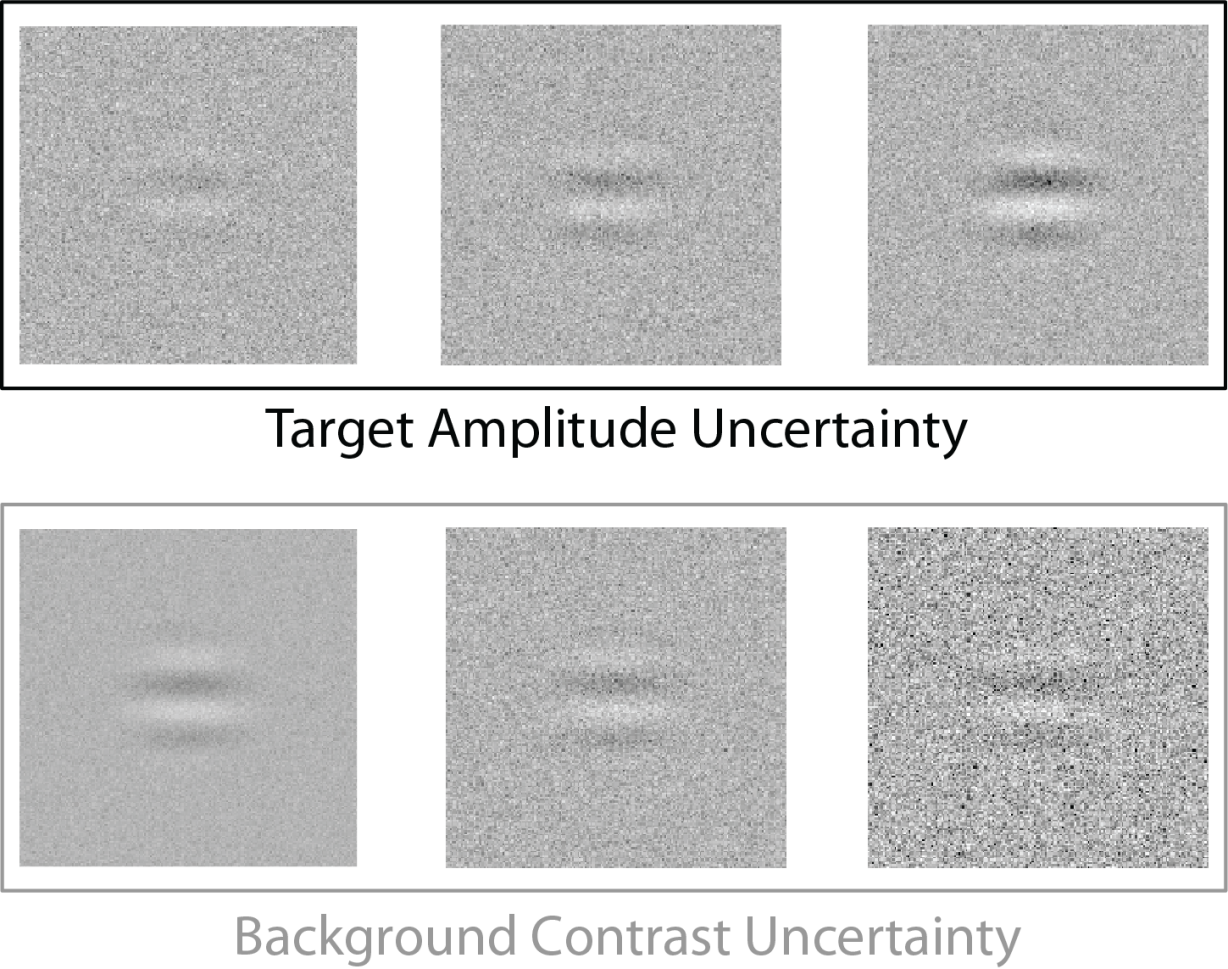
The example target was a sine wave windowed with a raised cosine. For target-present trials, the target amplitude was randomly sampled from multiple predefined levels that caused participants to be uncertain about the strength of the signal prior to detection (target-amplitude uncertainty). For every trial, background contrast was randomly sampled from multiple predefined levels that caused participants to be uncertain about the background contrast prior to detection (background-contrast uncertainty).

In Experiment 1, the task was to detect additive targets on white-noise backgrounds. In each target-absent trial, the background contrast was randomly sampled from a fixed predefined range of values (a prior probability distribution on background contrast). In each target-present trial, the target amplitude was also randomly sampled (independently) from a fixed predefined range of values (a prior probability distribution on target amplitude). Two conditions were run, one with a target-present prior of 0.5 and one with a target-present prior of 0.2. Target-amplitude uncertainty is illustrated in the upper panels of Figure 1, and background-contrast uncertainty in the lower panels. The detailed experimental methods are given in the next section. Here, we describe the four model observers for this task. We applied these same model observers to the task in Experiment 2, where the backgrounds were natural images rather than white noise.

### Model observers

**Figure 2.**
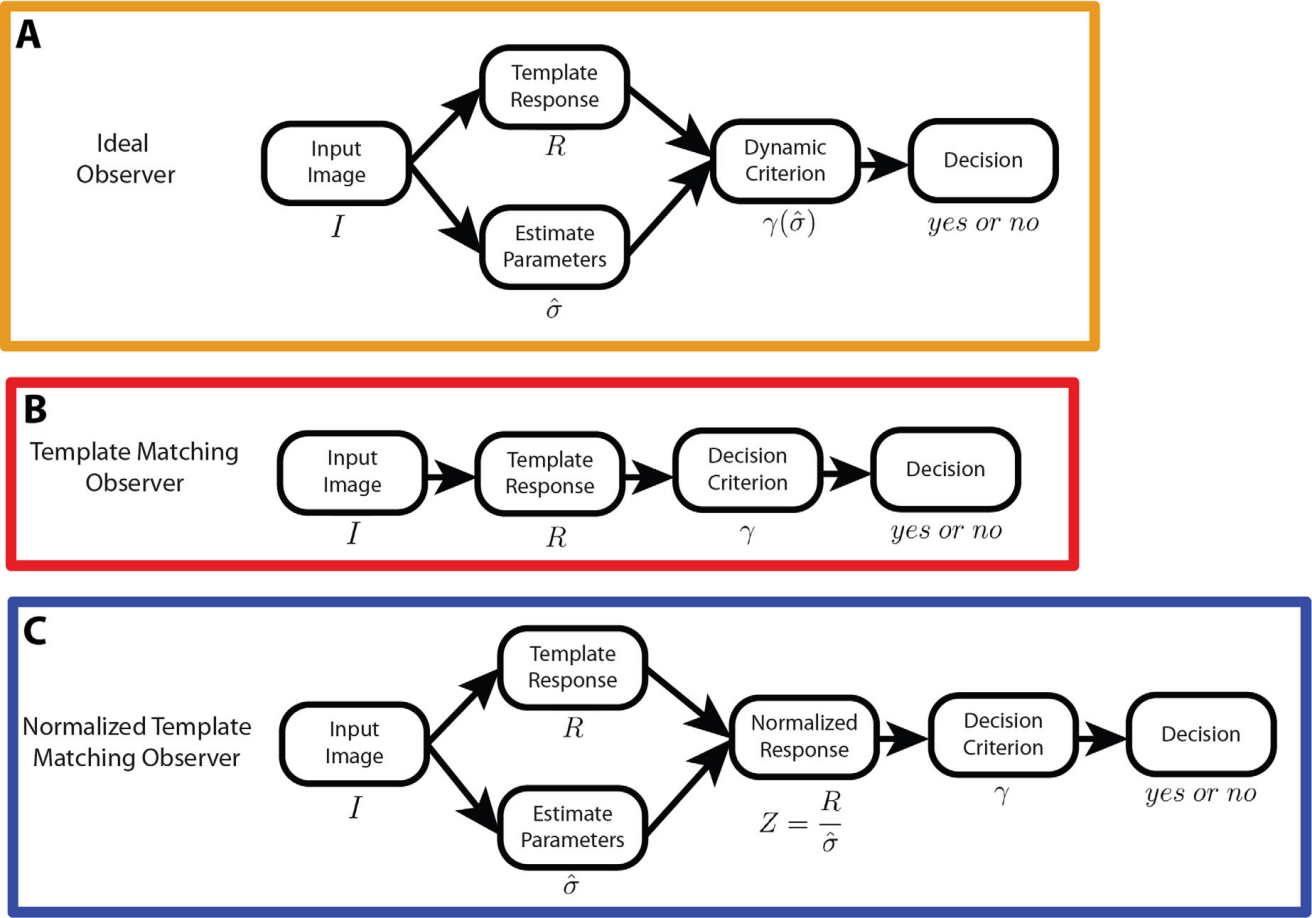
Three model observers. (**A**) The ideal observer was implemented with template matching and a dynamic criterion. In each trial, the template response was computed by taking the dot product of the template and the target region of the image. Also, the background contrast was estimated, and the template response was compared to a dynamic criterion based on the estimated background contrast. (**B**) The simple template-matching observer compared the template response to a single fixed criterion for all background contrast levels to produce a decision. (**C**) The normalized template-matching observer estimated the background contrast and calculated the standard deviation of template responses from it. Template response was normalized by the standard deviation of template responses for each background contrast level. The normalized response was compared to a single fixed criterion for all background contrast levels to produce a decision.

We start by describing the ideal observer (IO). We assume that the prior probability of the target being present and the prior probabilities of the target amplitudes and background contrasts are known to the ideal observer. Furthermore, we are assumed that the background contrast can be estimated with high precision on each trial from the background outside of the target region. Although this is true for white-noise backgrounds of the size in our experiment, it is less true for smaller white-noise backgrounds and for natural-image backgrounds because the contrast of the background is conflated with that of the target (Sebastian et al., 2017). The target location is also assumed to be known (no position uncertainty). The ideal observer responds “target present” if the posterior probability of target present is greater than that of target absent. We showed that the ideal observer can be implemented by extending a simple template-matching observer (for details, see Appendix A). In each trial, a simple template-matching observer computed the dot product of the template and the target region of the image and then made the decision by comparing the dot product (template response) to a criterion, γ. The template is defined to be the target, normalized by its total energy. If there were only amplitude uncertainty, comparing the template response to an optimally placed criterion would be equivalent to a maximum posterior estimation (the ideal observer) because the template response is a sufficient statistic for the likelihood ratio, and it is strictly increasing with the ratio (Appendix A). The optimal criterion depends on the background contrast; thus, the ideal observer can be implemented by comparing the template response to a dynamic criterion based on background contrast (Figure 2A).

The second model observer is the simple template-matching (TM) observer. Unlike the ideal observer, the TM observer compares template responses to a single fixed criterion for all contrast levels. The optimal single fixed criterion can be found via optimization (Figure 2B).

The third model observer is the normalized template-matching (NTM) observer. The NTM observer normalizes template response with the standard deviation of template responses (which is the background contrast scaled by a single constant). The normalized value is treated as a normalized template response and compared to a single fixed criterion (Figure 2C). The responses of neurons in the primary visual cortex are known to respond in a fashion consistent with contrast normalization (contrast gain control, Albrecht & Geisler, 1991; Heeger, 1992; Carandini & Heeger, 1994; Carandini et al., 1997; Cavanaugh et al., 2002). This normalization allows a simpler cognitive strategy (a single fixed criterion) that reasonably approximates the ideal observer (Sebastian et al., 2017).

The final model is a generalization of the ideal-observer model (Figure 2A). If dynamic criteria are chosen arbitrarily (optimal or non-optimal), we will call the model the dynamic template-matching (DTM) observer. We consider the more general DTM observer because humans undoubtedly have some ability to adjust their decision criteria but may not be able to do so in an optimal fashion.

### Simulations

All four model observers are image computable. However, instead of simulating model observers, we derived analytical expressions for hit and correct rejection rates to generate more precise predictions faster. The derivations can be found in Appendix A.

In all of the computational simulations and experiments, the target was a sine wave windowed with a raised cosine. The details of the target are described in the experimental methods below. The ranges of target amplitudes and background contrasts were picked to give approximately 75% correct in the psychophysical experiment. Luminance is reported in gray level values so that the same metric could be used for both amplitude and contrast. Gray values were linearized with a mean of 128 and a maximum of 255 (0 is the minimum). The amplitude of the target was randomly sampled from 50 logarithmically spaced and equally likely levels of target amplitude between 4 and 19 gray levels (the amplitude is defined to be the maximum gray level of the presented target, which corresponds to root mean square [RMS] contrast between 0.9% and 4% with a mean gray level of 128). The standard deviation of the Gaussian white noise was randomly sampled from 50 linearly spaced and equally likely levels between 5.12 and 12 gray levels. Corresponding RMS contrast of the backgrounds varied between 4% and 25%. We assumed that prior probability distributions of target amplitude and background contrast levels were uniform.

We compared the performance of the three model observers given the optimal criteria (picked to maximize the percentage correct) for various levels of an amplitude-range scalar—a single scalar that multiplies all of the amplitudes within the amplitude range. Here, we only present model predictions for when the amplitude range was scaled so the performance of three model observers was around 75% correct, when the probability of the target present was 0.5 (amplitude range scalar: 0.06). More detailed results of computational simulations (with other values of the amplitude scalar) can be found in GitHub page (https://github.com/CanOluk/Amplitude_Contrast_Uncertainty), but the results are generally similar.

**Figure 3.**
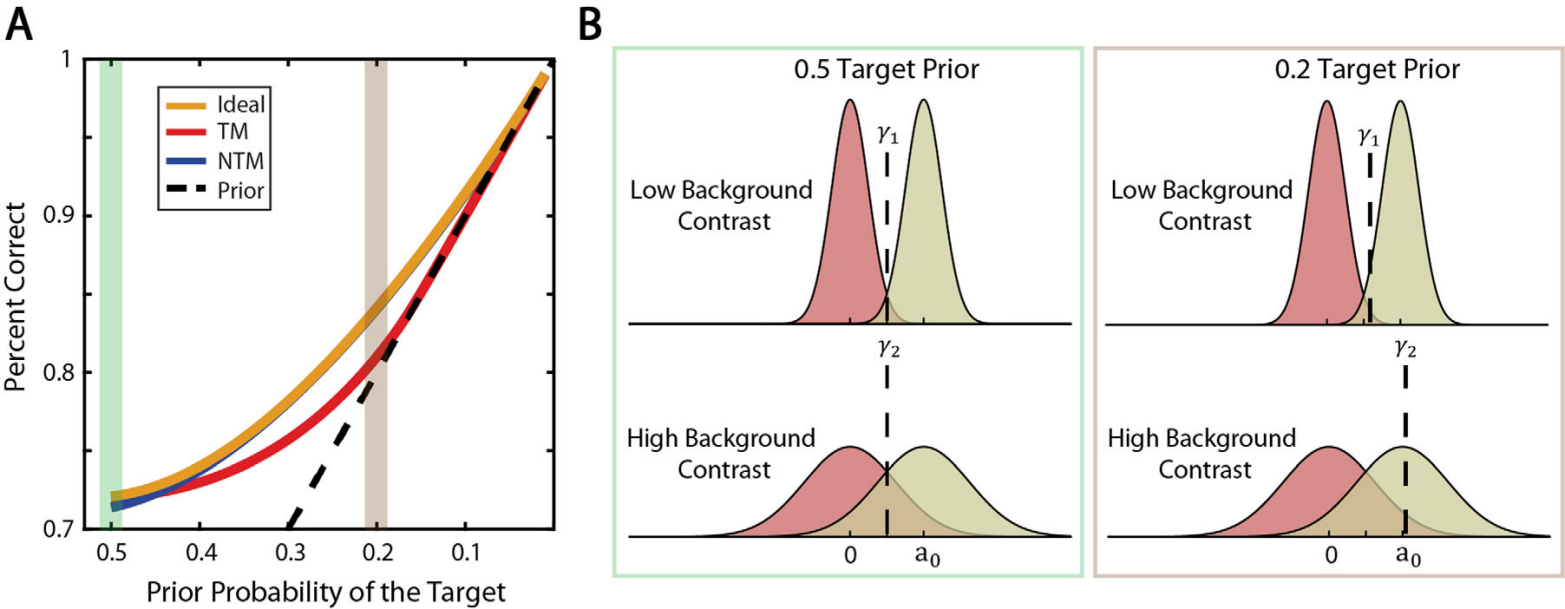
Simulation results. (**A**) The overall percent correct of each of the three model observers as a function of the prior probability of target being present is shown. The amplitude range was scaled down with a single scalar that gave approximately 75% correct when the target prior was 0.5 (threshold efficiency). When the target prior was low, the NTM observer well approximated the ideal observer and performed better than the TM observer, which was almost only as good as guessing based on prior probability only (shown with a dashed black line). (**B**) Illustration of the relationship between the ideal criterion and the target prior when there was only contrast uncertainty. The distribution of template responses when the target was absent is shown in red, whereas the yellow distribution corresponds to the case when the target was present. The mean of distributions when the target was present is equal to the target amplitude, and the standard deviation increased with background contrast linearly. When the target prior was 0.5, for all background contrast levels the optimal criterion was exactly the same as shown in the first panel; however, this was only a special case. In general, the optimal criterion (γ) depended on the standard deviation of the template responses, thus the background contrast.

We found that, for the target prior of 0.5, the overall percentage correct of the model observers was almost the same (Figure 3A). This occurred because the optimal criterion was approximately the same in all three models for different levels of background contrast. Consider the left panels in Figure 3B. The distribution of template responses is shown in red when the target was absent and shown in yellow when the target was present. The template responses for the target-present case have a mean equal to the target amplitude and a standard deviation that increases with the background contrast. If the target prior was 0.5 for all background contrast levels, the optimal criterion (producing the highest percent correct) was exactly the same: the target amplitude divided by two. However, this is not the case in general. If the prior probability of the target was different than 0.5, then the optimal criterion would be proportional to the standard deviation of template responses and, therefore, proportional to background contrast (see the right panels in Figure 3B). Consequently, one would expect that when the target prior was not 0.5 the predictions of the model observers would be more distinguishable, and the TM observer would perform worst.

A target prior of 0.5 is efficient for data collection in the laboratory, but target priors under natural conditions are almost always lower; thus, we simulated model performance for priors below 0.5. We found that for most of the low target priors, the NTM observer was better than the TM observer (Figure 3A). In general, the NTM observer well approximated the ideal observer, but the TM observer failed to do so when the target prior was low. When the target prior was 0.2, the NTM observer was better than the TM observer by 3% correct. We defined a relative performance measure that quantifies the importance of percent correct differences. The relative performance measure was calculated by scaling the performance such that the range between best performance (given by the ideal observer) and the baseline guessing performance (given by the prior probability) was scaled to a range of between 1 and 0. Therefore, the relative performance measure represents the fraction of the maximum performance increase from chance that the model observer captured. When the target prior was 0.2, the NTM observer captured more than 99% of the maximum increase, and the TM observer captured only 40% of the maximum increase. Thus, the TM observer was only slightly better than chance performance when the target prior was low.

**Figure 4.**
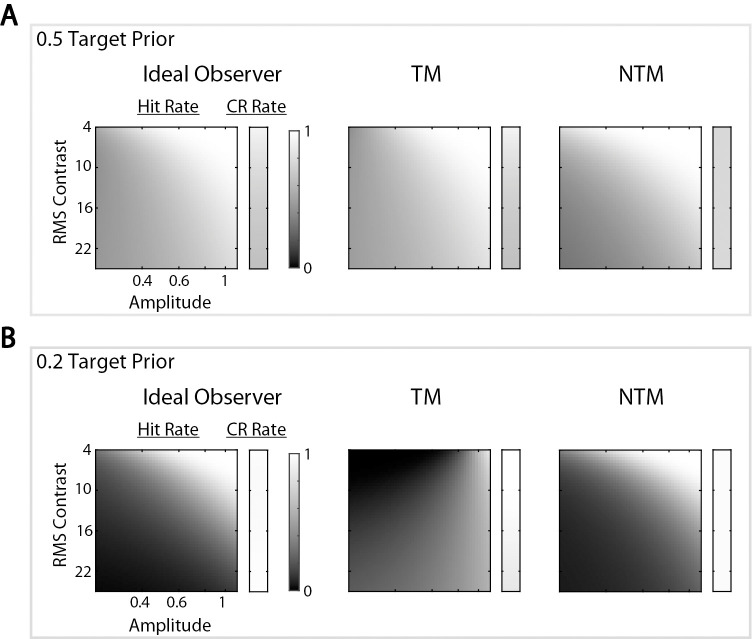
Hit and correct rejection rates of the three model observers. Model observers were simulated at threshold efficiency (as shown in Figure 3A). (**A**) Hit and correct rejection rates are shown when the target prior was 0.5 for 50 levels of target amplitude and background RMS contrast. The brightest white corresponds to a 100% hit (or correct rejection) rate, and the darkest black corresponds to 0%. For each level of background contrast, a correct rejection rate is shown in the smaller rectangle for each model observer. A 50-by-50 hit rate matrix is shown as the larger square for each model observer. Three model observers had similar hit and correct rejection rates. (**B**) Hit and correct rejection rates are shown when the target prior was 0.2. The TM observer had lower correct rejection rates when the background contrast was high, and the pattern of its hit rates is visibly different.

The overall percentage correct in the uncertainty experiment is a single measure of the performance. Even if two model observers have similar overall percentages correct, their computations and thus the hit and correct rejection rates might differ substantially. Examining these differences could be more efficient for model discrimination because it is relatively time consuming to run the same uncertainty experiment for enough target prior levels to measure a curve like that shown in Figure 3A. When the target prior was 0.5, we found that the simulated hit and correct rejection rates were similar for the three model observers (Figure 4A). However, when the target prior was 0.2, the TM observers’ predictions were quite different (Figure 4B). Thus, we expected that we should be able to distinguish between the TM and the other two model observers when the target prior was 0.2 but most likely would not be able to distinguish them when the target prior was 0.5. In the psychophysical experiment, we measured responses for the target prior levels of 0.5 and 0.2.

In sum, we described three model observers, including the ideal observer. Simulations revealed that the predictions of these model observers were very similar when the prior probability of the target was 0.5. However, for low target priors, the NTM observer approximated the ideal observer well, whereas the TM observer was closer to chance performance compared to the optimal performance. We showed that the TM observer should produce rather a different pattern of hits and correct rejections than the other two model observers.

## Methods

### Stimuli, subjects, and procedure

**Figure 5.**
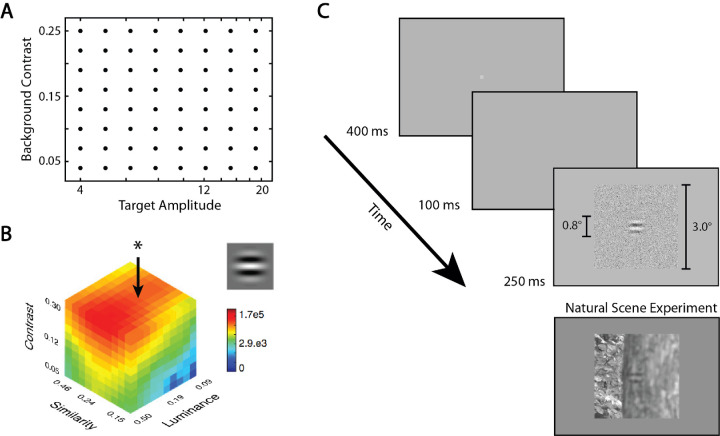
Experimental methods. (**A**) Target amplitude and background contrast levels in the white-noise experiment. For any target-present trial, the parameters of the stimulus were uniformly sampled from the grid of points. (**B**) Constrained sampling approach for the natural-scene backgrounds (adapted from Sebastian et al., 2017). Natural scenes are binned along three dimensions: luminance, RMS contrast, and amplitude spectrum similarity to the target presented (cosine wave, but the measure was phase invariant so it applied to a sine wave, as well). The star and arrow point out the column containing eight bins we used in the experiment with different mean RMS contrasts. The column corresponds to fixed median luminance and median similarity to the target. On each trial, a bin was randomly selected with uniform prior probability from eight bins to create background RMS contrast uncertainty. (**C**) The procedure of the experiment. The 400-ms fixation screen was followed by a 100-ms black screen. The stimulus was shown for 250 ms with a background of 3 visual degrees square. In the natural-scene background experiment, the background gray level was lower, but the procedure was the same. The example target-present stimulus presentation is shown.

The target was a 4-c/° horizontal sine wave, windowed with a radial raised-cosine function whose diameter was 0.8° at 120 pixels/°. The background was a square patch of white noise with a width of 3°. The target was presented at the center of the display together with the white-noise background. The stimuli and the experimental conditions were optimized for the psychophysical experiment. First, the white-noise background consisted of 4 × 4-pixel squares (2 × 2 minute), whose gray level was independently sampled from a Gaussian distribution.^1^ Using a virtual pixel size of 2 × 2 minute approximately matched the noise energy to the optics of the eye, thus allowing the noise to have a larger effect on performance. Second, there were only eight levels of target amplitude and background contrast instead of the 50 levels used in the simulations. We confirmed that the computational results extend to this sparser sampling. The eight logarithmically spaced levels of target amplitudes were 4, 5, 6, 8, 10, 12, 15, and 19 gray levels (with a mean gray level of 128, the corresponding RMS contrasts were 0.9%, 1.1%, 1.3%, 1.7%, 2.1%, 2.6%, and 4.0%). The eight levels of background gray levels were 5.12, 8.96, 12.80, 16.64, 20.48, 24.32, 28.16, and 32.00 (corresponding RMS background contrasts were 4%, 7%, 10%, 13%, 16%, 19%, 22%, and 25%) (Figure 5A).

We also conducted the same experiment with natural-scene backgrounds. Sebastian et al. (2017) binned millions of natural-image patches along three dimensions: mean luminance, RMS contrast, and amplitude-spectrum similarity to the target, all computed from the target region. On average, every natural background in a bin has almost the same luminance, contrast, and similarity to the target (with minor variations). We created RMS background contrast uncertainty by sampling across eight bins, each with a different mean RMS contrast. However, the luminance and similarity were held at their median value (sixth luminance bin and fifth similarity bin out of 10 bins along each dimension). The backgrounds had a mean gray level of 59. The standard deviations of the gray level of bins in the experiment were 3.25, 3.91, 6.83, 8.22, 9.90, 11.92, 14.35, and 17.28 (the corresponding RMS contrasts were 6%, 7%, 12%, 14%, 17%, 20%, 24%, and 29%). Target amplitudes were adjusted so that the average accuracy was approximately 75% correct. The amplitudes were 2, 3, 4, 5, 7, 9, 13, and 17 gray levels (corresponding RMS contrasts were 0.9%, 1.4%, 1.8%, 2.3%, 3.2%, 4.1%, and 7.8%). Each background was sampled from appropriate bins without replacement, so participants never saw the same background twice. We made sure that the target with the smallest amplitude contained at least four different discrete gray levels and that the discretization has negligible effects on model observers’ discriminability.

The stimuli were presented at a distance of 171 cm with a resolution of 120 pixels/°. In the white-noise experiment, the mean luminance of the background was 44.87 cd/m^2^. For natural backgrounds, the mean luminance was 20.7 cd/m^2^. Images were gamma corrected based on the calibration of the display device (GDM-FW900; Sony, Tokyo, Japan) and quantized to 256 gray levels. The screen refresh rate was 85 hertz. All experiments and analyses were done using custom code written in MATLAB (MathWorks, Natick MA), using the Psychophysics Toolbox (Brainard, 1997; Pelli, 1997).

Four experienced observers completed the experiment with the white-noise backgrounds, and three of them completed the experiment with natural-scene backgrounds. They had normal (corrected) spatial acuity. Written, informed consent was obtained for all observers in accordance with The University of Texas at Austin Institutional Review Board.

The procedures in both experiments were the same. Participants were asked to press a key to deliver their decision about whether the target was present or absent in each trial. Each trial started with a dim fixation point at the center, presented for 400 ms, followed by a 100-ms blank window. The stimulus was presented for 250 ms (Figure 5C). Participants were given 1 second to indicate their decision. If a participant failed to respond in this time window, the trial was excluded from the analysis. The number of excluded trials was less than 1% of the total trials. The subsequent trial began after an additional 500 ms. The stimulus presentation time was selected to match the average human fixation duration during a visual search. A high-frequency feedback tone was provided if the participant's response was correct, and a low-frequency if the response was incorrect. On each trial, the target amplitude and the background contrast were sampled randomly from the 64 possible combinations (see Figure 5A); however, the sampling was counterbalanced so each condition had the same number of trials.

In each block of the experiment, the prior probability of the target was either 0.5 or 0.2, and participants were informed before starting the block. Every participant completed three blocks for each target prior condition (one participant only joined the white-noise experiment and completed two blocks). The order of the blocks was randomized. In a single block, there was a total of 640 trials. When the target prior probability was 0.5, the number of trials for each unique combination of contrast and amplitude level (total of 64 conditions in which the target was present) was 5. When the target prior probability was 0.2, it was 2.

### Analysis

The simulation methods were adjusted to the changes we made for the psychophysical experiment (see above and Appendix A). All three models were fitted to the trial-by-trial data using a maximum-likelihood method. For each participant, and for their combined data, we estimated three parameters for the TM and NTM observers. The first parameter was a scaling parameter on the target amplitudes, which allows for an overall efficiency difference between the model and participants. The other two parameters were a separate criterion for each target prior. For the DTM observer, there was a single scaling parameter, but the criterion was allowed to vary with background contrast. We also calculated the predictions of the ideal observer (DTM observer with optimal criteria) for the amplitude scalar factor estimated for the DTM observer. The ideal observer's criteria were completely determined by the background contrast; hence, there were no free parameters except the amplitude scale factor. The parameters were estimated independently for the white noise and natural image backgrounds. Confidence intervals were generated by resampling the data and fitting the bootstrapped data. Results in the upcoming sections are reported for the average observer, which was generated by aggregating all of the participants’ data (individual participant's results can be found in the appendices and the GitHub page). More detailed analysis methods can be found in Appendix A. In a separate analysis described in Appendix C, we allowed the scaling parameter to vary with target prior probability but found that the added parameter had little effect.

## Results

### White noise backgrounds

**Figure 6.**
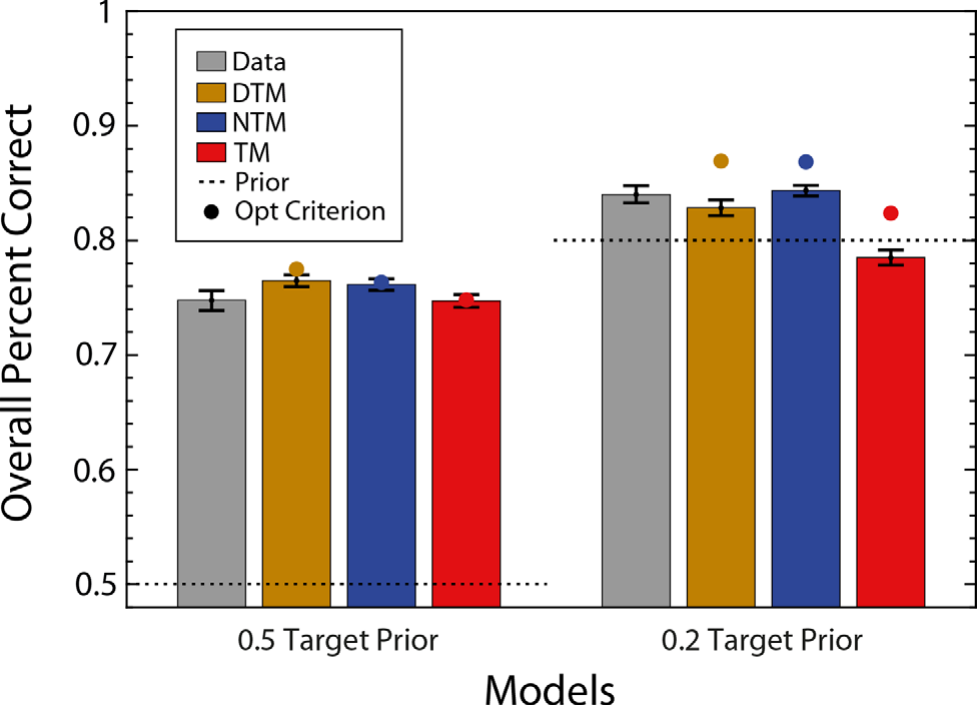
Overall percent correct for human and model observer in the white-noise experiment. Gray bars show the overall percent correct of the average participant. The average participant's data were generated by aggregating all of the participants’ data. The colored bars show the predictions of three model observers whose parameters were estimated from fitting the detailed pattern of hits and correct rejections (see Figure 7). The left set of bars is for the high target prior condition and the right for the low target prior. The colored dots show the model predictions when the decision criteria were adjusted to maximize accuracy while holding the amplitude-scalar parameter fixed. The dashed lines indicate the prior of target absent.

The gray bars in Figure 6 show the overall percent correct of the average participant for the two target prior conditions. As expected, accuracy is higher when the prior differed from 0.5. The colored bars show the accuracy of the model observers for the parameter values estimated from the maximum-likelihood fitting. The colored dots show the accuracy when the decision criteria were adjusted to provide the maximum possible accuracy (without changing the amplitude scaling factor). As can be seen, the accuracy of the DTM and NTM observers was almost identical and a bit higher than that of the TM observer. The fitted TM observer's accuracy matched human accuracy for the 0.5 prior and was substantially below for the 0.2 prior.

The fitted DTM and NTM observers’ accuracies were slightly above human accuracy for the 0.5 priors and straddled human accuracy for the 0.2 prior. Overall, the DTM and NTM observers gave a slightly better fit to average human accuracy, although the DTM observer did have a greater number of free parameters.

**Figure 7.**
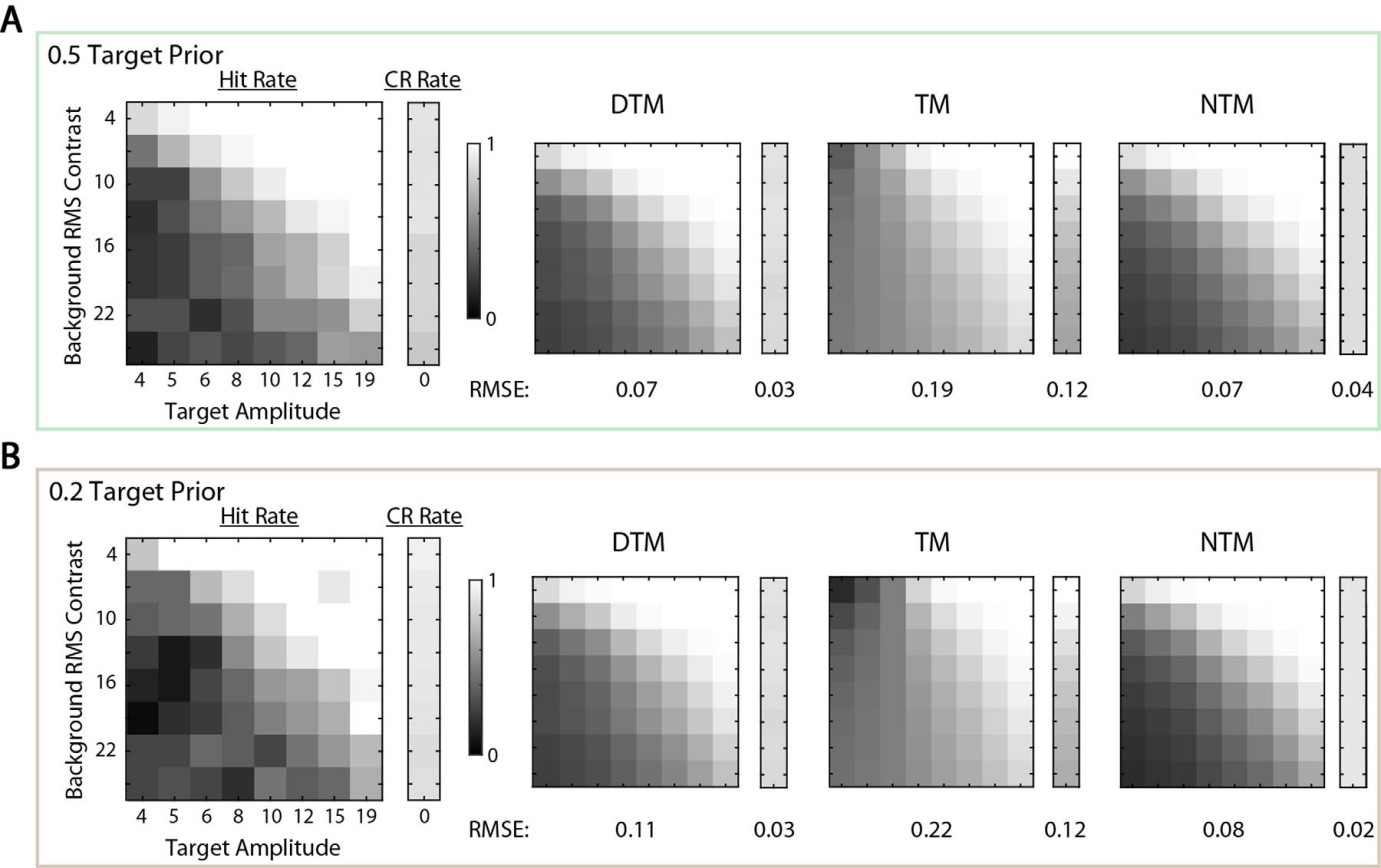
Results of white-noise experiment. (**A**) The data matrix is an eight-by-eight hit rate matrix of the average participant followed by a column of correct rejection rates when the prior probability of the target present was 0.5. (**B**) The data matrix of the average participant when the target prior was 0.2. Grayscale is used to cover rates between 1 (white) and 0 (black). Best-fit model observer hit and correct rejection rates are presented in the same structure. For the fitted estimates, root-mean-squared errors (RMSEs) were calculated for hit and correct rejection matrices, and they are presented below the corresponding matrices. The negative likelihoods and 68% confidence intervals associated with the DTM, TM and NTM observers are 10,784 (10,628–10,903), 12,699 (12,559–12,831) and 10,836 (10,693–10,968). respectively. Scale factors associated with the DTM, TM, and NTM observers are 0.41, 0.35, and 0.41, respectively.

More substantial differences among the model observers are revealed by considering the detailed predictions for the hits and correct rejections as a function of target amplitude and background contrast. Correct rejection and hit rates for the average participant are shown in the left panel of Figure 7A for the target prior of 0.5 and in the left panel of Figure 7B for the target prior of 0.2. Model predictions are shown in the three right panels. Surprisingly, we found that, even for the target prior of 0.5, the TM observer failed to explain the pattern of hit and correct rejection rates, although it did produce a similar overall percent correct (Figure 6). As expected, when the target prior was 0.2, the NTM and DTM observers better explained the data than the TM observer (Figure 7B).

**Figure 8.**
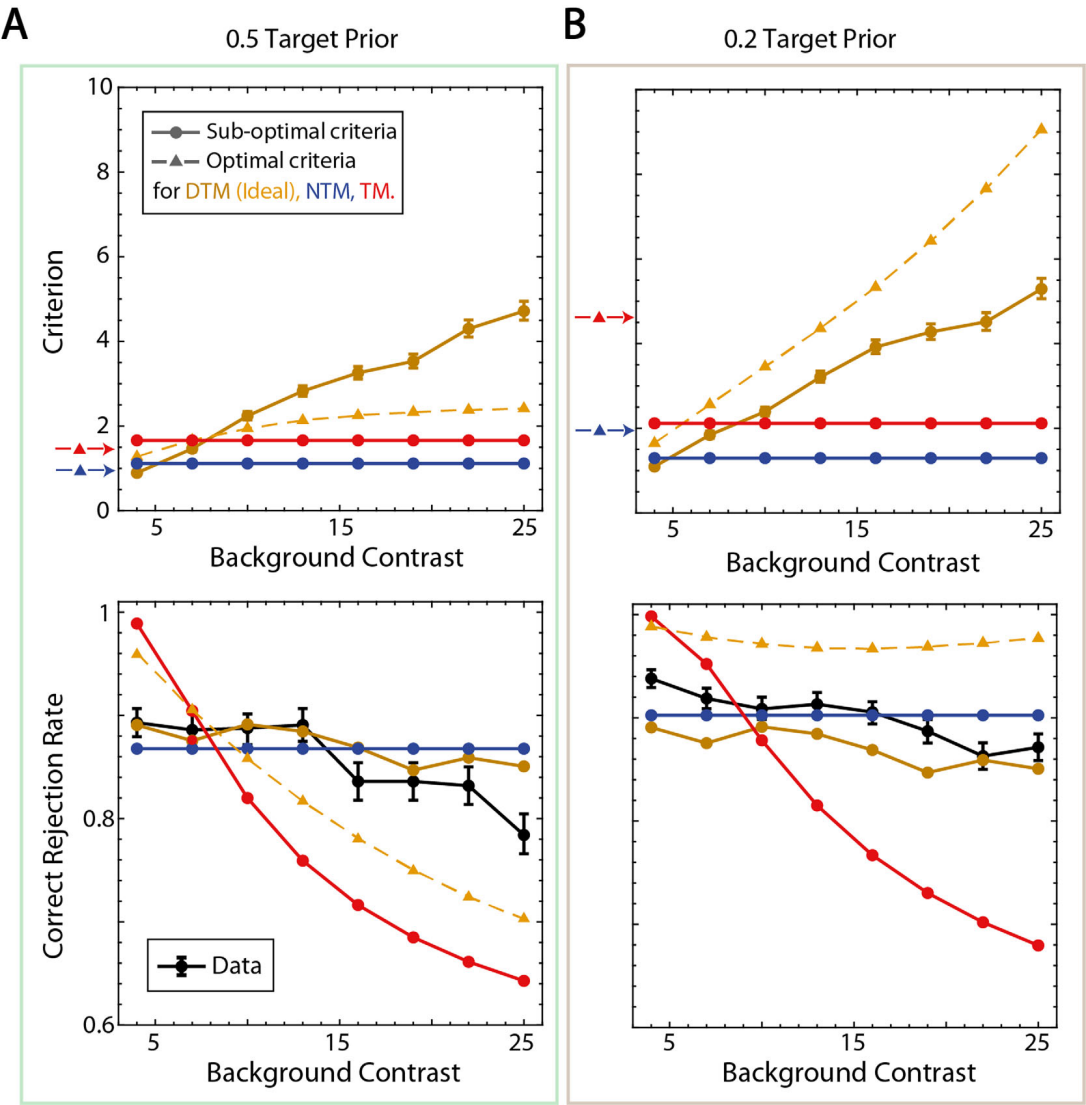
Decision criteria and correct rejection rate. (**A**) Estimated criteria for three model observers associated with fits to the average participant and correct rejection rates when the target prior was 0.5. Error bars show the 68% confidence intervals. For each model observer, given the estimated scale factor, the optimal criteria are shown as dashed lines and arrows (DTM becomes the ideal observer with optimal criteria shown in yellow). Correct rejection rates of the average participant are shown in black with 68% confidence intervals. (**B**) Estimated criteria for three model observers associated with fits to the average participant and correct rejection rates when the target prior was 0.2.

Note that the fitted TM observer's predictions were qualitatively different from the predictions shown in Figure 4 when the target prior was 0.2. Specifically, the orientation of the iso-hit-rate contours differed. This difference occurred because the best-fit criterion was very non-optimal, as shown later (see Figure 8), whereas the predictions in Figure 4 are for an optimal criterion.

Overall, the NTM and DTM observers better explained the data for both target conditions than the TM observer. The root-mean-squared error (RMSE) between predictions and the data are given below the plots of hit and correct rejection rates in Figure 7. More rigorously, the figure caption gives the negative likelihoods and 68% confidence intervals for the three model observers. The likelihoods are higher for the NTM and DTM observer, and their confidence intervals do not overlap the confidence interval of TM observer. The results also show that measuring the detailed pattern of hit and correct rejection rates can be a powerful approach to discriminating among model observers.

**Figure B1. figA21:**
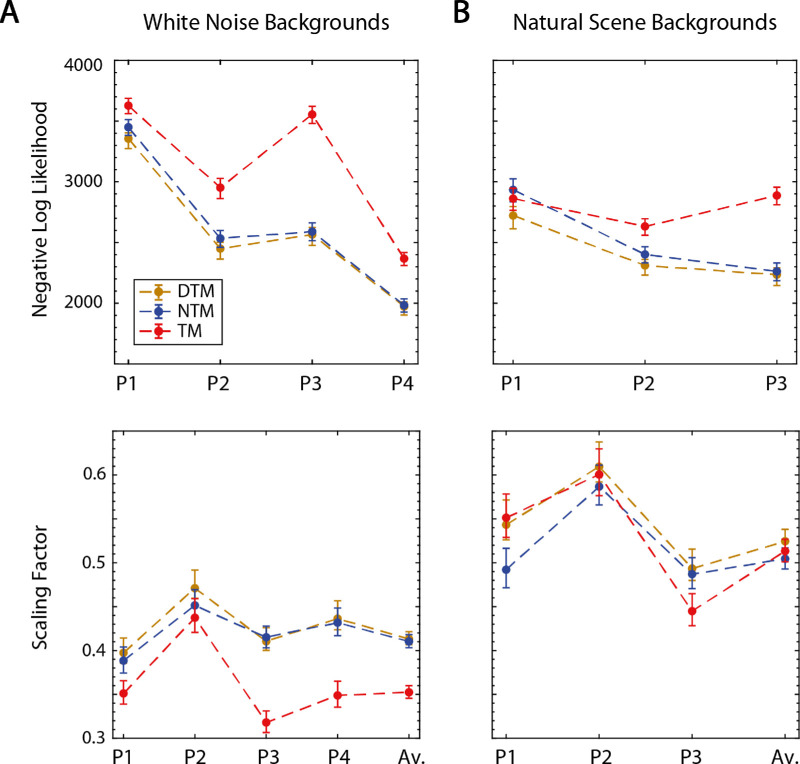
Goodness of fits and scale factors. (**A**) For the white-noise background experiment, the goodness-of-fit measure (negative log likelihood) and the estimated scaling factor for the individual participants (“Av.”) is used to label the average participant. Error bars show the 68% confidence intervals. The confidence intervals show that the TM observer consistently failed to explain the data as well as the NTM and DTM observers across participants. The estimated scaling factors mostly fall in the range of 0.4 to 0.3 and are relatively stable across participants. (**B**) For the natural-scene background experiment, the goodness-of-fit measure (negative log likelihood) and the estimated scaling factor for the individual participants are shown. The TM observer failure was still consistent across participants, but this time the NTM observer also seemed to explain the data slightly worse than the DTM observer (for the first participant, it was even worse than the TM observer). The estimated scaling factors had more variability and were slightly higher than in the white-noise experiment, which is not surprising considering that template matching for natural scenes is not the ideal strategy. Also, the TM observer had estimated scaling factors similar to those of the DTM and NTM observers, unlike in the white-noise experiment.

**Figure C1. figA31:**
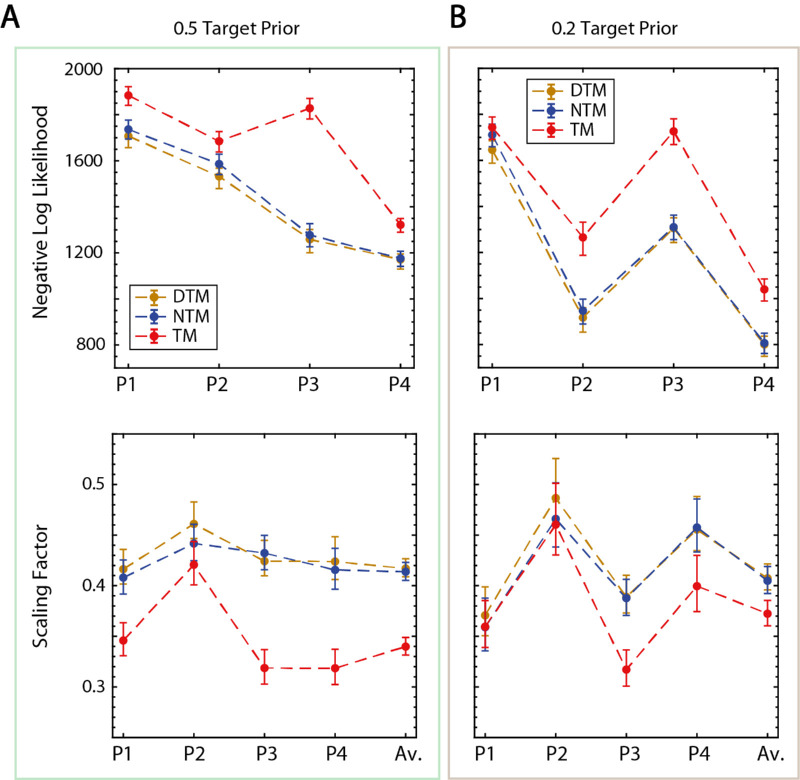
Goodness of fits and scale factors for the white-noise backgrounds experiment when the scale factor was allowed to vary with prior condition. (**A**) Goodness-of-fit measure (negative log likelihood) and estimated scaling factor for individual participants when the target prior was 0.5 are shown. “Av.” is used to label the average participant. Error bars show the 68% confidence intervals. For the average participant, the negative log likelihoods and 68% confidence intervals associated with the DTM, TM, and NTM observers were 5922 (5827–6000), 6832 (6750–6911), and 5940 (5851–6023), respectively, for the 0.5 target prior. (**B**) Goodness-of-fit measure (negative log likelihood) and estimated scaling factor for individual participants when the target prior was 0.2 are shown. For the average participant, the negative log likelihoods and 68% confidence intervals associated with the DTM, TM, and NTM observers were 4861 (4746–4955), 5862 (5746–5970), and 4896 (4787–4998), respectively. Thus, the DTM and NTM observer better explained the average data for both target priors than the TM observer. The TM observer's failure was consistent across all participants, and the estimated scalar factors for each target prior were also comparable.

**Figure C2. figA32:**
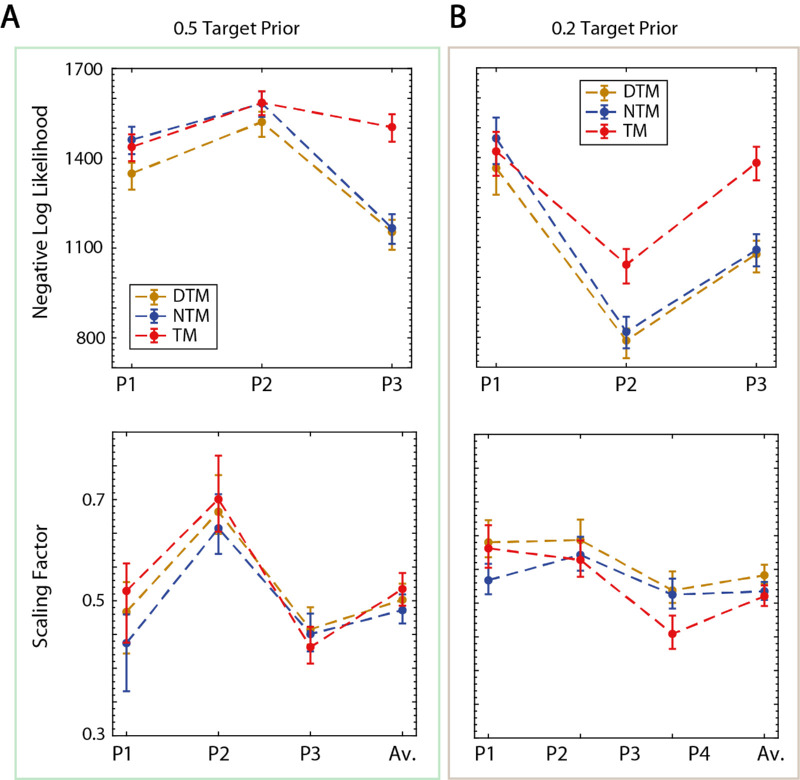
Goodness of fits and scale factors for the natural-scene background experiment when the scale factor was allowed to vary with prior condition. (**A**) Goodness-of-fit measure (negative log likelihood) and estimated scaling factor for individual participants when the target prior was 0.5 are shown for the natural-scene background experiment. “Av.” is used to label the average participant. Error bars show the 68% confidence intervals. For the average participant, the negative log likelihoods and 68% confidence intervals associated with the DTM, TM, and NTM observers were 4227 (4138–4298), 4629 (4552–4702), and 4368 (4283–4445), respectively, for the 0.5 target prior condition. (**B**) Goodness-of-fit measure (negative log likelihood) and estimated scaling factor for individual participants when the target prior was 0.2 for the natural-scene background experiment. For the average participant, the negative log likelihoods and 68% confidence intervals associated with the DTM, TM, and NTM observers were 3351 (3228–3454), 3892 (3770–4007), and 3438 (3320–3551), respectively. Similarly, the DTM and NTM observers accounted for the data better than the TM observer. The TM observer's failure was consistent across all participants, and the estimated scalar factors for each target prior were also comparable. In sum, allowing the scaling factor to vary across the target prior conditions had a negligible effect on the results. The failure of the TM observer was due to the pattern of hits and correct rejections it can produce. Thus, it cannot be resolved by simply increasing overall performance.

The results were robust across participants. The TM observer consistently failed to explain the data as well as the NTM and DTM observers across participants (except for one participant when the target prior was 0.2; see Appendix B). In Appendix B (Figure B1A), we show that the estimated scaling factors mostly fall in the range of 0.35 to 0.45 and were relatively stable across participants. The scaling factors estimated for the DTM and NTM observers are similar (0.41 for the average participant), but the best-fit scaling factor for the TM observer is slightly smaller (0.35 for the average participant). We also show in Appendix C that, even if the scale factor is allowed to be different for the low and high prior conditions, the best estimates of the scale factors are nearly identical (Figures C1 and C2). The small variation in scaling factors shows that the scaling factor represents an overall efficiency difference between human participants and model observers. Thus, the scaling factor has a negligible impact on the pattern of correct rejection and hit rates.

Both the NTM and DTM observers explained the data equally well; however, the DTM observer used eight criterion parameters, whereas the NTM observer used only one. We calculated standard model selection measures to penalize for the number of parameters and found that, for Bayesian information criteria (BIC), the NTM observer was slightly better than the DTM observer on average, but the confidence intervals mostly overlap, so they still explained the data equally well. We also checked whether using a dynamic criterion after the normalization would improve the fit of the NTM observer. However, adding the dynamical criterion made the NTM observer the same as the DTM observer and provided no extra explanatory power. Also, no explanatory power gain was apparent from the estimated criteria for the dynamic criterion NTM observer because it had only a small variation (almost completely flat criteria). Finally, we compared the BIC of the three model observers to the BIC calculated with an empirical model that uses the empirical correct rejection and hit rates (8 + 64 parameters). We found that, for the average participant, the TM observer was worse than the empirical model; however, both the DTM observer and the NTM observer were better than the empirical model.

The difference among the three model observers is perhaps best illustrated by the correct rejection rates for the different levels of background contrast. Correct rejection rates are an immediate result of the criteria because all distributions of template responses for target-absent conditions sit at zero (see Figure 3). The circles in the upper panels in Figure 8 show the estimated decision criteria as a function of background contrast for the three model observers; the triangles show the decision criteria of the ideal observer. The criteria for the TM and NTM observers (red and blue circles) were fixed and could not vary with background contrast. The criteria for the best fitting DTM observer (dark yellow circles) increased monotonically with background contrast, with about the same slope for the 0.5 and 0.2 prior conditions. The criteria for the ideal observer (yellow triangles) also increased monotonically, but with a steeper slope for the 0.2 prior condition. The red and blue triangles to the left of each panel show the values of the criterion that maximized the overall accuracy of the TM and NTM observers (for the given scaling factor); see also colored dots in Figure 6.

The black circles in the lower panels in Figure 8 show that the average participant's correct rejection rates decreased slowly and are close to flat. However, the fitted TM observer's correct rejections fell rapidly with background contrast. The NTM observer had a flat correct rejection rate; nonetheless, its predictions were still quite good and explain the data just about as well as the DTM observer (especially given the greater number of free parameters for the DTM observer). The correct rejection rates of the ideal observer (IO) are shown in dashed yellow with triangles. Note that, when the prior was 0.5, it was similar to the TM observer in terms of the slope; however, when the target prior was 0.2, it was much flatter and thus similar to the NTM observer.

### Natural-scene backgrounds

**Figure 9.**
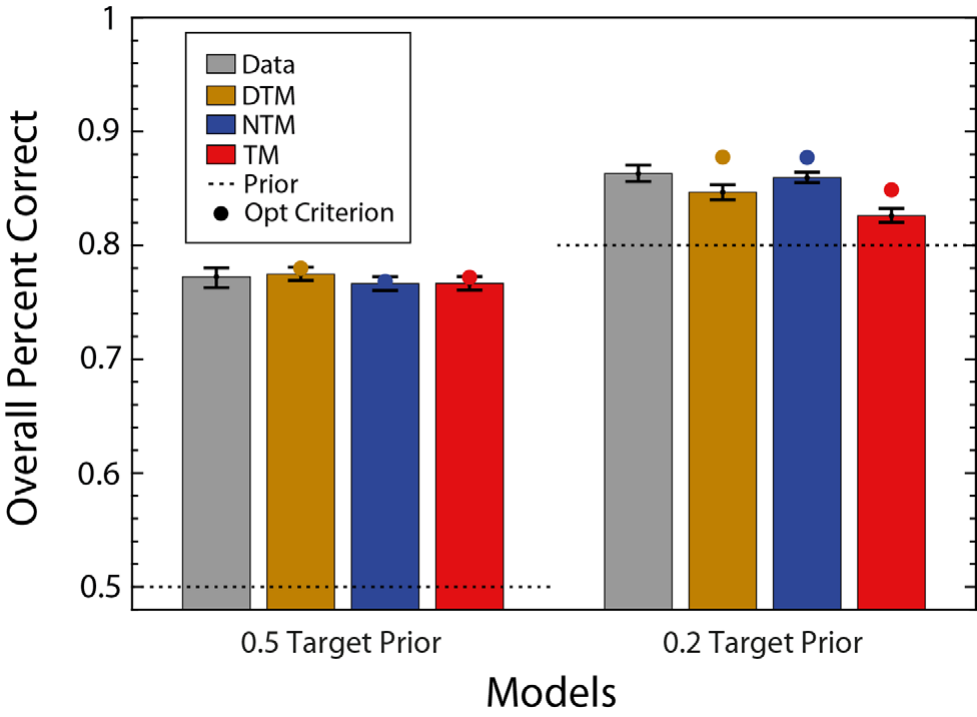
Overall percent correct for human and model observers in the natural-scene background experiment. Gray bars show the overall percent correct of the average participant. The colored bars show the predictions of the three model observers whose parameters were estimated from fitting the detailed pattern of hits and correct rejections (see Figure 10). The left set of bars is for the high target prior condition and the right for the low target prior. The colored dots show the model predictions when the decision criteria were adjusted to maximize accuracy while holding the amplitude-scalar parameter fixed. The dashed lines indicate the prior of target absent.

**Figure 10.**
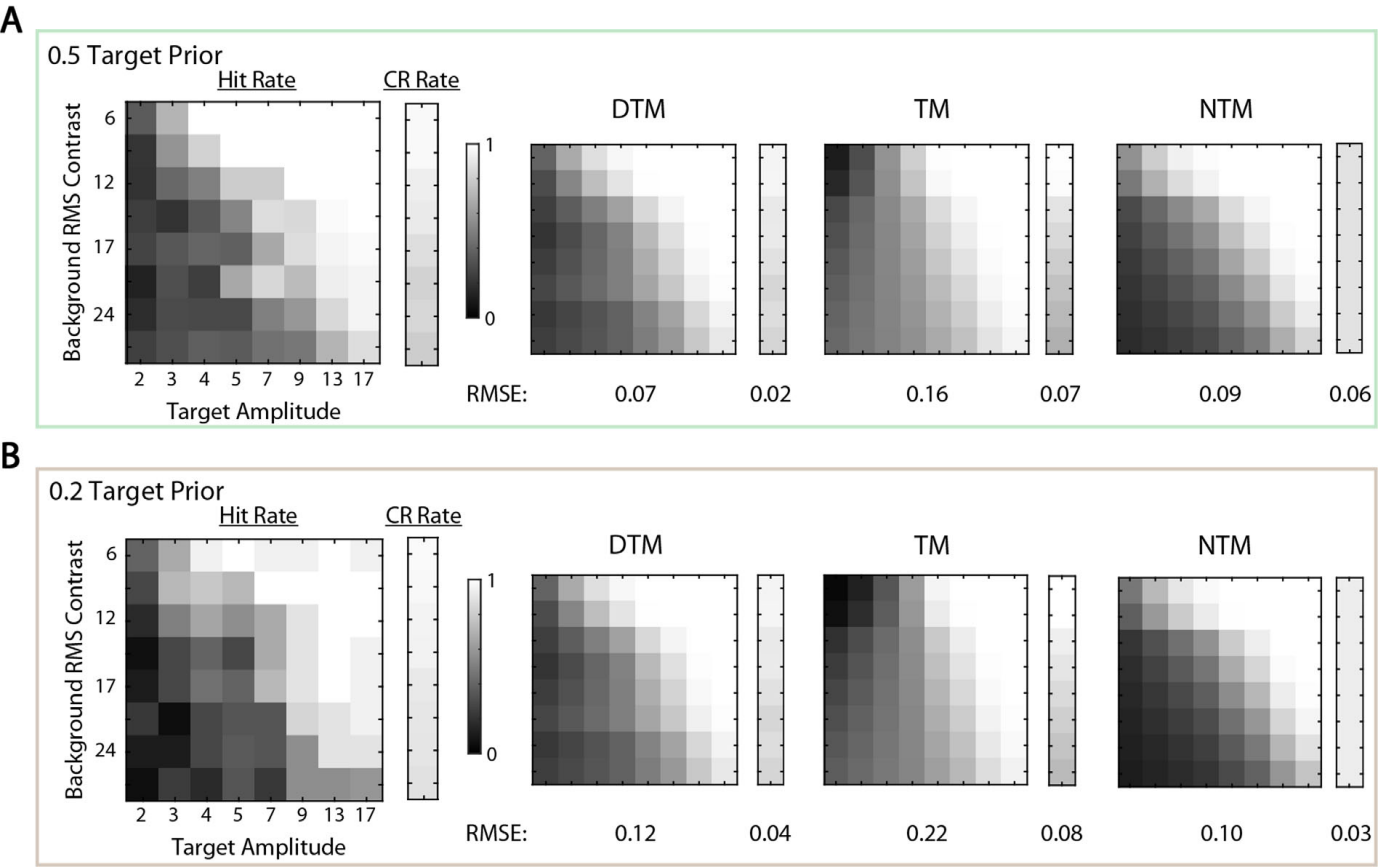
Results of the natural-scene experiment. (**A**) The data matrix is an eight-by-eight hit rate matrix of the average participant followed by a column of correct rejection rates when the prior probability of the target present was 0.5. (**B**) The data matrix of the average participant when the target prior was 0.2. Grayscale is used to cover rates between 1 (white) and 0 (black). Best-fit model observers’ hit and correct rejection rates are presented in the same structure. For the fitted estimates, RMSEs were calculated for hit and correct rejection matrices, and they are presented below the corresponding matrices. The negative likelihoods and 68% confidence intervals associated with the DTM, TM, and NTM observers are 7581 (7427–7701), 8521 (8380–8658), and 7807 (7665–7943), respectively. Scale factors associated with the DTM, TM, and NTM observers are 0.52, 0.51, and 0.50, respectively.

The results are almost the same for the natural-scene backgrounds. All three model observers could explain the overall percent correct when the target prior was 0.5, and the DTM and NTM observers did a slightly better job when the target prior was 0.2 (Figure 9). The hit and correct rejection rates are shown in Figure 10, together with model fits. Similar to the white-noise experiment, the DTM and NTM observers better fit the hit and correct rejection rates than did the TM observer for both target priors. This result is consistent across participants, but this time it was clearest with a target prior of 0.2 (see Appendix B). In contrast, when the target prior was 0.5, the NTM observer seemed to explain the data slightly worse than the DTM observer (and for one participant it explained the data slightly worse than the TM observer). The estimated scaling factors were more variable and slightly higher than in the white-noise experiment, which is not surprising given the greater statistical variability of natural backgrounds and that template matching for natural backgrounds is not the ideal strategy. The scaling factor for the TM observer was similar to those for the DTM and NTM observers, unlike in the white-noise experiment.

Also, we found again that, when the scale factor was allowed to be different for the low and high prior conditions, the best estimates of the scale factors were nearly identical, and so the predictions remained nearly the same (see Appendix C). Again, this is consistent with the hypothesis that human performance differs from model observer performance by a single overall scale factor.

**Figure 11.**
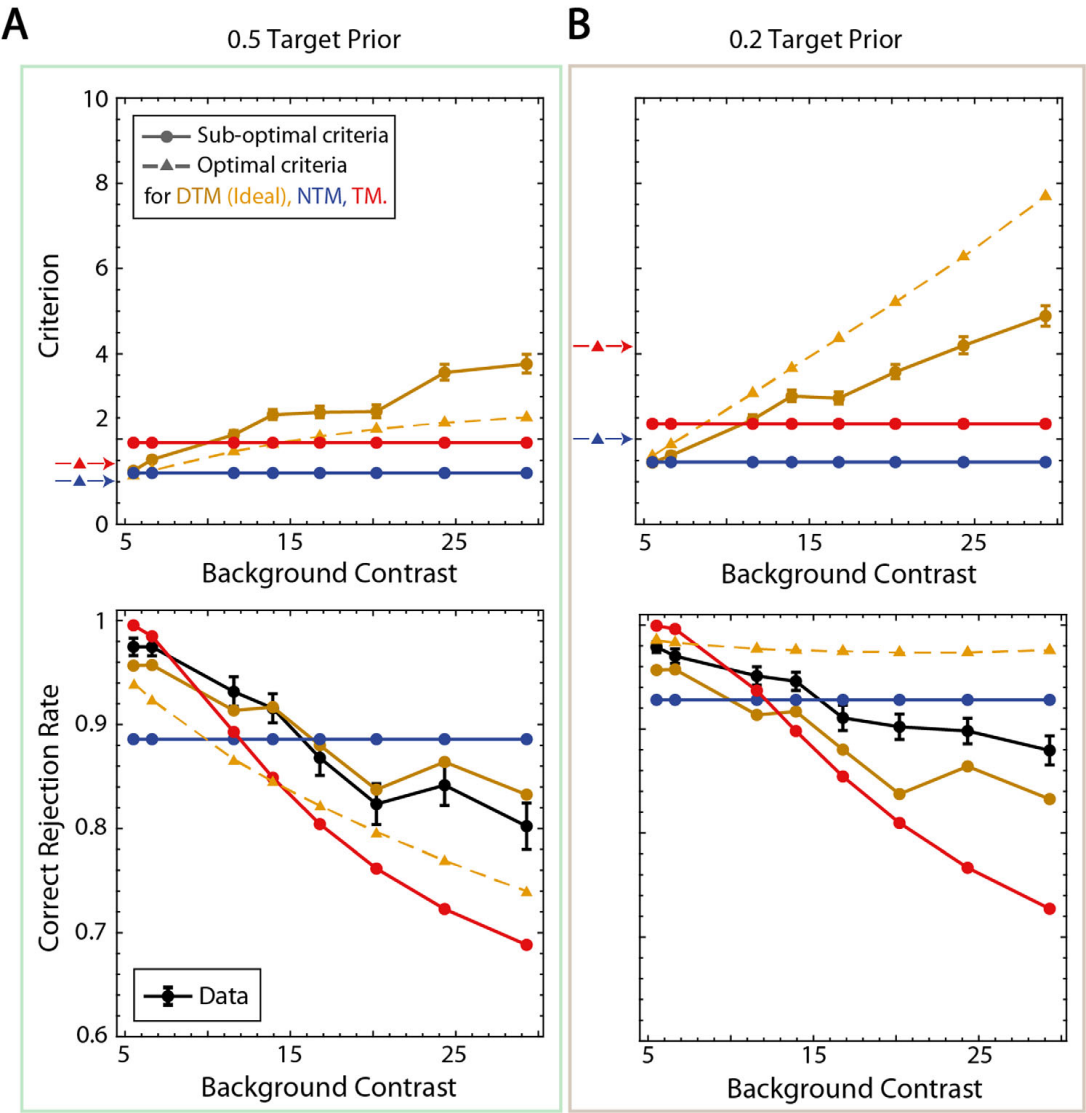
Criteria and correct rejection rates. (**A**) Estimated criteria for three model observers associated with fits to the average participant and correct rejection rates when the target prior was 0.5 for the natural-scene background experiment. Error bars show the 68% confidence intervals. For each model observer, given the estimated scale factor, optimal criteria are shown in dashed lines and arrows (the DTM observer becomes the ideal observer with optimal criteria shown in yellow). Correct rejection rates of the average participant are shown in black with 68% confidence intervals. (**B**) Estimated criteria for three model observers associated with fits to the average participant and correct rejection rates when the target prior was 0.2 for the natural-scene background experiment.

The upper panels of Figure 11 show the estimated criteria of the model observers for the average participant, and the lower panels show the correction rejection rates of the average participant and model observers. The patterns of parameter values and correct rejection rates are similar to that for white-noise backgrounds (Figure 8). One modest difference is that the slope of the estimated criterion plot for the DTM observer is shallower than for white noise. This difference is closely tied to the steeper falloff in human correct rejection rate with background contrast. In order to predict the steeper falloff, the DTM observer must keep its criterion more constant. This steeper falloff in the correction rejection rate is the reason why the NTM observer's RMSE is greater than that of the DTM observer for the 0.5 prior condition (see Figures 8 and 10). Nonetheless, we found that the NTM and DTM observers were indistinguishable when we calculated the BIC. Furthermore, the NTM and DTM observers did better than the default model, which used the empirical correct rejection and hit rates to predict the data.

## Discussion

Most research on the effects of extrinsic stimulus uncertainty has focused on one or two stimulus dimensions at a time. Here, we measured and modeled the joint effect on target detection performance of random target amplitude, background contrast, and background pattern across trials. For such cases, the standard approach of comparing performance with and without uncertainty is difficult or impractical, because of the number of experimental trials required. Therefore, we explored a new approach that instead examines the detailed pattern of hits and correct rejections as a function of target amplitude, background contrast, and prior probability of target present, under large uncertainty about target amplitude, background contrast, and background pattern.

We compared human performance with three model observers: simple template-matching (TM), normalized template-matching (NTM), and dynamic template-matching (DTM). All three model observers were only slightly affected by target-amplitude variability alone (in agreement with previous psychophysics; e.g., see Davis et al., 1983), although the TM observer was more strongly affected when there was simultaneous background-contrast variability. The TM observer was strongly affected by background-contrast variability. The NTM observer was less affected by background-contrast variability because it normalized the template responses on each trial by the estimated contrast (see https://github.com/CanOluk/Amplitude_Contrast_Uncertainty). Similarly, the DTM observer was less affected by background-contrast variability because it used the estimated background contrast to shift its decision criterion on each trial. A special case of the DTM observer is the ideal observer (in white noise), which shifted the criterion optimally on each trial and hence was not affected by the background-contrast variability.

We found that the human pattern of hits and correct rejections, in white noise and natural backgrounds, was predicted relatively well and much better by the NTM and DTM observers than by the TM observer or the ideal observer. In simulations, we showed that discriminating among the model observers is, in principle, easier by using a prior probability of target present that is less than 0.5. Although this is generally true, in the current study we found that it was also possible to discriminate well among the models even when the prior probability of the target present was 0.5. Nonetheless, our results suggest that there may be substantial benefits of varying prior probabilities for discriminating among models. Our results also demonstrate the potential value of studying the effects of extrinsic variability with the efficient method of measuring the detailed pattern of hits and correct rejections in a single block, where all of the dimensions of variability being studied are simultaneously and independently randomly sampled on each trial.

### The mechanisms of visual detection under uncertainty

The TM observer represents the null hypothesis that no additional mechanisms, other than looking for the target's features, are required to explain the human visual detection performance under simultaneous target amplitude and background-contrast uncertainty. However, we show that it consistently failed to explain the data, even though the TM observer was still better than humans in its absolute performance. The results of the current experiment are consistent with two possible mechanisms for dealing with stimulus uncertainty: normalization and dynamic criterion. In the primate visual system, there is strong neurophysiological evidence for contrast normalization (Heeger, 1992; Albrecht & Geisler 1991), as well as normalization along other stimulus dimensions (Carandini & Heeger, 1994; Carandini et al., 1997; Cavanaugh et al., 2002). Moreover, the inclusion of normalization in image-processing models can explain psychophysical data under various conditions (Watson & Solomon, 1997; Sebastian et al., 2017). The current results, as well as the simulations here and in Sebastian et al. (2017), show that normalization is particularly useful when the prior probability of a target being present is low, as it is under many natural conditions. Thus, the NTM observer is a parsimonious and plausible explanation for the current results.

There are also biologically plausible implementations of dynamic criteria. First, there might be built-in mechanisms, learned through evolutionary history, that are automatically applied when making decisions about object detection under different levels of background contrast. Specifically, it is possible that signals carrying information about the visibility of the target, such as background contrast, automatically alter the decision criteria, which would be consistent with a previous finding showing that the decision criteria can be modulated based on confidence about the perceptual decision in a following memory task (Solomon, Cavanagh, & Gorea, 2012). Second, participants might learn the relationship between background contrast and criteria during the experiment. It is clear that participants can cognitively keep multiple criteria in mind. Indeed, widespread experimental methods (rating scales) depend on this ability. One possible disadvantage of this strategy is that keeping multiple criteria in mind might hurt performance. For example, cognitive load due to keeping multiple criteria in mind could decrease performance compared to performance in experiments that do not require multiple criteria (Egan, Schulman, & Greenberg, 1959; Swets, Tanner, & Birdsall, 1961; Benjamin, Tullis, & Lee, 2013; Tekin & Roediger, 2017). However, an eight-point scale has been shown not to affect the measured detection performance (Swets, 1959). Therefore, participants in the current experiment might be cognitively selecting on each trial from something like eight criteria positioned based on the estimated background contrast level.

Although this may be plausible under the conditions of the current experiment, it is less plausible under real-world conditions where targets are often randomly located over a substantial region of the visual field. It is very unlikely that decision criteria are dynamically adjusted with each new fixation at all relevant image locations, especially via cognitive control. Normalization, on the other hand, is applied automatically in parallel across the visual field. Thus, the most plausible hypothesis is that normalization is the dominant mechanism underlying the effects of uncertainty (even in the current experiment), with a modest task-dependent contribution of dynamic criterion adjustment.

## Conclusions

The visual system almost always faces multiple simultaneous dimensions of extrinsic uncertainty in the real world. We measured human performance for detecting an additive sine-wave target in white noise and natural backgrounds, when background contrast and target amplitude are randomly varying over a wide range for different prior probabilities of target present (0.2 and 0.5). We found that human performance was consistent with two possible mechanisms for dealing with stimulus uncertainty: contrast normalization and dynamic criterion. Considering previous evidence for contrast normalization (gain control) in the cortex and the implausibility of having multiple criteria for every stimulus level, the visual system most likely exploits contrast normalization to perform efficiently under uncertainty. In general, our results show the value of contrast normalization under simultaneous target amplitude and background contrast variability and the value of having these mechanisms for performing visual detection under natural conditions, which almost always include uncertainty along these dimensions.

In this study, unlike previous studies, we focused solely on the high uncertainty condition, and we examined hit and correct rejection rates to distinguish between model observers. Our results demonstrate the usefulness of this approach. We also showed that manipulating prior probability could be a more informative strategy than previously thought for understanding the behavior of model observers (and the visual system) and for distinguishing among model observers, particularly for complex naturalistic tasks. In future studies, it should be possible to extend the current approach to more naturalistic conditions by combining the current dimensions of uncertainty with additional common dimensions of uncertainty.

## Footnotes

1 The target was not downsampled to 4 × 4-pixel squares; thus, the exact ideal observer would have exploited the difference between samplings. However, it is very unlikely that predictions of the exact ideal observer would be any different.

## Appendix A. Appendix A: Single criterion is optimal under amplitude uncertainty

We assumed that the prior probability of the target being present and the prior probabilities of target amplitudes and background contrasts are known to the ideal observer. Furthermore, the ideal observer is assumed to know the background contrast because the background patch is big enough to estimate it precisely. The target location is also assumed to be known (no position uncertainty). When the background contrast is known to the observer, the problem reduces to making an ideal decision under amplitude uncertainty because the appropriate ideal observer can be evaluated for each background contrast level. The ideal observer responds “target present” if the ratio of the likelihood of target present (*H*_1_) to absent (*H*_0_) is more than the ratio of the prior probability of target absent to present:

$$\frac{P\left(I\;|\;H_{1}\right)}{P\left(I\;|\;H_{0}\right)}>\frac{P\left(H_{0}\right)}{P\left(H_{1}\right)}$$

Ω*_s_* is the target region, and *I_x_* is the gray level of the location *x* in the image. When the target is present, luminance at any location is the sum of the target gray level (*T_x_*) and the gray level of the Gaussian noise background (mean of 0 and standard deviation of σ*_n_*) that is sampled independently for each location. In a target-present trial, only one of the amplitudes (*a_m_*) is used, so they are disjoint events and exhaustive. Therefore, the likelihood can be expressed as a sum of the probabilities of these disjoint events. The likelihood of target-present and target-absent are given by

$$P\left(I\;|\;H_{1}\right)=\sum_{m}\left(\prod_{x\in \Omega_{s}}G\left(I_{x}\;|\;a_{m}T_{x},\sigma_{n}\right)\right)P\left(a_{m}\right)$$

$$P\left(I\;|\;H_{0}\right)=\prod_{x\in \Omega_{s}}G\left(I_{x}\;|\;0,\sigma_{n}\right)$$

Explicitly writing out Gaussian distributions leads to

$$\frac{P\left(I\;|\;H_{1}\right)}{P\left(I\;|\;H_{0}\right)}=\sum_{m}e^{\frac{a_{m}}{\sigma_{n}^{2}}\;\sum_{x\in \Omega_{s}}\left(I_{x}T_{x}\right)}\;e^{\frac{-a_{m}^{2}}{2\sigma_{n}^{2}}\;\sum_{x\in \Omega_{s}}{\left(T_{x}\right)}^{2}}\;P\left(a_{m}\right)$$

In the experiment, every term was fixed except the dot product of the target region with the target ($\sum_{x\in \Omega_{s}}\left(I_{x}T_{x}\right)$). Therefore, the dot product is a sufficient statistic for the likelihood ratio (captures all the information in the likelihood ratio). Also, the ratio increases monotonically with the dot product; therefore, a single optimal criterion on this metric would be equivalent to the maximum posterior estimation. There are no further simplifications for the derivation of the optimal criterion, and its value depends on the background contrast. Therefore, the ideal observer can be implemented by comparing the dot product to a dynamic criterion that changes based on the background contrast level.

### Mean and standard deviation of template responses

For a given pair of target amplitude and background contrast, it is possible to derive the mean and standard deviation of distributions of dot products ($\sum_{x\in \Omega_{s}}\left(I_{x}T_{x}\right)$). If the target is present and amplitude is *a_m_*, then the gray level of the image at any point (*I_x_*) is distributed as Gaussian with a mean of *a_m_T_x_* and standard deviation of σ*_n_*. The dot product is also distributed as Gaussian with a mean of $a_{m}\sum_{x\in \Omega_{s}}T_{x}^{2}$ and standard deviation of $\sigma_{n}\sqrt{\sum_{x\in \Omega_{s}}T_{x}^{2}}$.

$$\;\;\;\;\;\;\;\;\;\;\;\;\;I_{x}∼G\left(a_{m}T_{x},\sigma_{n}\right)$$

$$\;\;\;\;\;\;\;\;\;\;\;\;\;I_{x}T_{x}∼G\left(a_{m}T_{x}^{2},\sigma_{n}T_{x}\right)$$

$$\;\;\;\;\;\;\;\;\;\;\;\;\;\sum_{x\in \Omega_{s}}\;I_{x}T_{x}∼G\;\left(\;a_{m}\sum_{x\in \Omega_{s}}\;T_{x}^{2},\sqrt{\sum_{x\in \Omega_{s}}\;{\left(\sigma_{n}T_{x}\right)}^{2}}\;\right)\;\left(\mathrm{Variances}\;\mathrm{add}\right)$$

If the target is absent, the gray level of the image at any point (*I_x_*) is distributed as Gaussian with a mean of 0 and standard deviation of σ*_n_*. The dot product is also distributed as Gaussian with a mean of 0 and standard deviation of $\sigma_{n}\sqrt{\sum_{x\in \Omega_{s}}T_{x}^{2}}$.

A template is generated by normalizing the target with a constant (*k*). Note that these operations only change the location of the optimal criterion and do not change the result presented above about a single criterion being optimal. In our case, we normalized the target by the energy of the target to generate a template.

$$Tm_{x}=\frac{T_{x}}{k}$$

$$k=\sum_{x\in \Omega_{s}}T_{x}^{2}$$

The mean and standard deviation of template responses can be derived from the mean and standard deviation of dot products by dividing them by the normalization constant. Distributions of template responses, if the target is present, are Gaussian with means of *a_m_* and standard deviations of $\sigma_{n}\frac{1}{\sqrt{\sum_{x\in \Omega_{s}}T_{x}^{2}}}$. The distribution of template responses, if the target is absent, is a Gaussian with mean of 0 and standard deviation of $\sigma_{n}\frac{1}{\sqrt{\sum_{x\in \Omega_{s}}T_{x}^{2}}}$.

### Analytical derivations of hit and correct rejection rates for three model observers

Instead of simulating model observers, we derived analytical expressions for hit and correct rejection rates to generate more precise predictions faster. First, consider the ideal observer (IO and DTM) implemented with a dynamic criterion. We begin by deriving the hit (or correct rejection) rate for a given amplitude and background contrast pair because these pairs are disjoint and exhaustive. Thus, the overall hit (or correct rejection) rate is the sum over amplitude and contrast levels with respect to their prior probabilities. For each amplitude and background contrast pair, in the case of white-noise background and additive targets, the distribution of template responses (*R_b_* + *a_i_*) (template response can be divided into response to background and response to target) is Gaussian. Means correspond to the amplitude levels of the target (*a_i_*, 0 if the target is absent). Standard deviations (σ*_j_*) are background contrasts (standard deviation of white noise) scaled by a constant. Note that the exact correspondence depends on how the template is defined in relation to the target as shown above. A model observer makes a hit if the template response is higher than the criterion and the target is present. Therefore, the overall hit rate of the ideal observer can be derived by integrating the probability of template response exceeding the criterion for every target-present condition. For each background level, there is a unique criterion (γ*_j_*).

$$\begin{array}{ccc} \;\;\;\;\;\;\;\;\;p_{h}\left(\gamma_{j}\right) & \;= & \sum_{j}\sum_{i}p\left(R_{b}+a_{i}>\gamma_{j}\;\left|\;a_{i},\sigma_{j}\right.\right)p\left(a_{i}\right)p\left(\sigma_{j}\right)\\ \;\;\;\;\;\;\;\;\; & \;= & \sum_{j}\sum_{i}\Phi \left(\frac{a_{i}-\gamma_{j}}{\sigma_{j}}\right)p\left(a_{i}\right)p\left(\sigma_{j}\right) \end{array}$$

where Φ(*x*) is the standard normal integral function. The model observer makes a correct rejection if the template response is lower than the criterion and the target is not present. The correct rejection rate of the ideal observer can be derived by integrating the probability of template responses that do not exceed the criterion for every target-absent condition. Because all target-absent distributions have an equal mean of 0, integration over the different contrast levels gives the correct rejection rate.

$$\begin{array}{ccc} p_{cr}\left(\gamma_{j}\right) & \;= & \sum_{j}p\left(R_{b}<\gamma_{j}\;\left|\;0,\sigma_{j}\right.\right)p\left(\sigma_{j}\right)\\ & \;= & \sum_{j}\Phi \left(\frac{\gamma_{j}}{\sigma_{j}}\right)p\left(\sigma_{j}\right) \end{array}$$

The optimal criteria can be found via optimization using these equations.

Second, we consider the simple template-matching (TM) observer. Unlike the ideal observer, simple template matching compares template responses to a single fixed criterion for all contrast levels. The hit and correct rejection rates of the TM observer can be expressed similarly, and the optimal single fixed criterion can be found via optimization.

$$\begin{array}{ccc} \;\;\;\;\;\;\;\;\;p_{h}\left(\gamma \right) & \;= & \sum_{j}\sum_{i}p\left(R_{b}+a_{i}>\gamma \;\left|\;a_{i},\sigma_{j}\right.\right)p\left(a_{i}\right)p\left(\sigma_{j}\right)\\ \;\;\;\;\;\;\;\;\; & \;= & \sum_{j}\sum_{i}\Phi \left(\frac{a_{i}-\gamma }{\sigma_{j}}\right)p\left(a_{i}\right)p\left(\sigma_{j}\right) \end{array}$$

$$\begin{array}{ccc} \;\;\;\;\;\;\;\;\;p_{cr}\left(\gamma \right) & \;= & \sum_{j}p\left(R_{b}<\gamma \;\left|\;0,\sigma_{j}\right.\right)p\left(\sigma_{j}\right)\\ \;\;\;\;\;\;\;\;\; & \;= & \sum_{j}\Phi \left(\frac{\gamma }{\sigma_{j}}\right)p\left(\sigma_{j}\right) \end{array}$$

Finally, we consider the normalized template-matching (NTM) observer. The NTM observer normalizes template response with the standard deviation of template responses (which is the background contrast scaled by a single constant). The normalized value is treated as a normalized template response $\left(Z_{b}+\frac{a_{i}}{\sigma_{j}}\right)$ and compared to a single fixed criterion. The standard deviations of the template responses (for all amplitude and contrast levels) are scaled to be 1 by the normalization. However, the mean is the initial mean divided by the standard deviation of the template responses $\left(\frac{a_{i}}{\sigma_{j}}\right)$. Given the mean and standard deviation, the hit rate can be written in a similar form.

$$\begin{array}{ccc} \;\;\;\;\;\;\;\;\;\;p_{h}\left(\gamma \right) & \;= & \sum_{j}\sum_{i}p\left(Z_{b}+\frac{a_{i}}{\sigma_{j}}>\gamma \;\left|\;\frac{a_{i}}{\sigma_{j}},1\right.\right)p\left(a_{i}\right)p\left(\sigma_{j}\right)\\ & \;= & \sum_{j}\sum_{i}\Phi \left(\frac{a_{i}}{\sigma_{j}}-\gamma \right)p\left(a_{i}\right)p\left(\sigma_{j}\right) \end{array}$$

To calculate the correct rejection rate, integration over these equally likely identical normal Gaussian distributions can be simplified in a single-step computation.

$$p_{cr}\left(\gamma \right)=\sum_{j}p\left(Z_{b}<\gamma \;|\;0,1\right)p\left(\sigma_{j}\right)=\Phi \left(\gamma \right)$$

### Details of simulation adjustments and model fitting

The simulation methods were adjusted to changes we made for the psychophysical experiment. The background contrast and target amplitude levels were the same as those used in the psychophysical experiment (only eight levels each). For 4 × 4-pixel white noise, the relationship between target amplitude and the mean of template responses was estimated by computing template responses for numerous samples of 4 × 4-pixel noise. We found that the mean of the distribution equals the target amplitude (as expected). The exact estimation was done to determine the relationship between the standard deviation of white noise and the standard deviation of template responses. Even though the standard deviation of templates responses is still a scaled version of the standard deviation of noise by a single constant (0.14), the constant is approximately four times the one analytically derived for a single-pixel white noise. These estimated relationships were used to calculate the percentage correct with the equations provided above.

For natural-scene backgrounds, three model observers’ hit and false-alarm rates must be calculated. We calculated the distribution of template responses for each natural-scene bin used in the experiment for target-present and target-absent conditions. We found that the distribution of template responses is well approximated with a Gaussian distribution consistent with earlier findings (Sebastian et al., 2017). The mean of these distributions is the target amplitude, and the standard deviation is the scaled background RMS contrast with a single scalar (0.13). Because these are Gaussians with known means and standard deviations, the same equations can be used to integrate the distributions, so the same fitting procedure can be applied.

We decided to fit the correct rejection rates (for the different background contrast levels) and hit rates (for the different pairs of background contrast and target amplitude levels) using a maximum-likelihood method. In each trial, the participant's decision was a hit, false alarm, correct rejection, or miss (depends on whether or not the target was presented). Thus, all data can be written as a string of hits, false alarms, correct rejections, and misses. Model observers predict the probabilities of these four categories as their hit and correct rejection rates. The probability of the entire data is the product of the corresponding probabilities of each response (or element in the string of data). This method is a variation of the classic maximum-likelihood method for fitting a psychometric curve to the data. We extended it for the data collected in the uncertainty experiment because it better deals with properties of the data (number of trials being different for different conditions and high or low rates likely to be more consistent) than standard metrics such as a squared error. Confidence intervals were generated by resampling the data assuming the response in a trial followed a binomial distribution with a success rate of corresponding hit (miss) and correct rejection (false alarm) rates. Confidence intervals for fitted parameters were computed by fitting the bootstrapped data.

## Appendix B. Appendix B: Individual participants

 [Fig figA21]

## Appendix C. Appendix C: Different scale factors for different priors

 [Fig figA31][Fig figA32]
